# Avoid or seek light – a randomized crossover fMRI study investigating opposing treatment strategies for photophobia in migraine

**DOI:** 10.1186/s10194-022-01466-0

**Published:** 2022-08-11

**Authors:** Eva Matt, Tuna Aslan, Ahmad Amini, Kardelen Sariçiçek, Stefan Seidel, Paul Martin, Christian Wöber, Roland Beisteiner

**Affiliations:** 1 Imaging-Based Functional Brain Diagnostics and Therapy, Department of Neurology, High Field Magnetic Resonance Centre, Medical University of Vienna, Spitalgasse 23, 1090 Vienna, Austria; 2 Department of Neurology, Medical University of Vienna, Vienna, Austria; 3 School of Applied Psychology, Griffith University, Southport, QLD Australia; 4 Department of Psychiatry, Monash University, Victoria, Australia

**Keywords:** Migraine, Photophobia, Habituation, Headache, Functional magnetic resonance imaging

## Abstract

**Background:**

Photophobia, the aberrantly increased sensitivity to light, is a common symptom in migraine patients and light discomfort is frequently found as a trigger for migraine attacks. In behavioral studies, planned exposure to light was found to reduce headache in migraine patients with photophobia, potentially by increasing habituation to this migraine trigger. Here, we aimed to elucidate neurophysiological mechanisms of light exposure versus light deprivation in migraine patients using functional magnetic resonance imaging (fMRI).

**Methods:**

Ten migraine patients (9 female, age = 28.70 ± 8.18 years) and 11 healthy controls (9 female, age = 23.73 ± 2.24 years) spent one hour on 7 consecutive days exposed to flashing light (Flash) or darkness (Dark) using a crossover design with a wash-out period of 3 months. Study participants kept a diary including items on interictal and ictal photophobia, presence and severity of headache 7 days before, during and 7 days after the interventions. One week before and one day after both interventions, fMRI using flickering light in a block design was applied. Functional activation was analyzed at whole-brain level and habituation of the visual cortex (V1) was modeled with the initial amplitude estimate and the corrected habituation slope.

**Results:**

Mean interictal photophobia decreased after both interventions, but differences relative to the baseline did not survive correction for multiple comparisons. At baseline, flickering light induced activation in V1 was higher in the patients compared to the controls, but activation normalized after the Flash and the Dark interventions. V1 habituation indices correlated with headache frequency, headache severity and ictal photophobia. In the Flash condition, the individual change of headache frequency relative to the baseline corresponded almost perfectly to the change of the habituation slope compared to the baseline.

**Conclusions:**

On average, light exposure did not lead to symptom relief, potentially due to the short duration of the intervention and the high variability of the patients’ responses to the intervention. However, the strong relationship between visual cortex habituation and headache symptoms and its modulation by light exposure might shed light on the neurophysiological basis of exposure treatment effects.

**Trial registration:**

NCT05369910 (05/06/2022, retrospectively registered).

## Background

Migraine is a highly prevalent neurological disorder with recurrent headache attacks, frequently accompanied by hypersensitivity to various sensory stimuli. Photophobia, an intolerance to the exposure to light, is the most frequent associated symptom. During the attack (ictally), literally all individuals with migraine report increased sensitivity to light [1]. Interictal photophobia is observed in up to 74% of people suffering from migraine [1], particularly in the premonitory and the postdrome phases [2, 3]. Photophobia has been shown to correlate with age, depressive symptoms, anxiety and stress [4].

Exposure to flickering light for instance, is also reported as a trigger for migraine attacks. Up to 69% of the patients identify visual discomfort as a precipitating factor for their attacks [5]. The standard advice to avoid migraine attacks has been to identify and to avoid triggers. However, there is no strong empirical support for the benefit of trigger avoidance and the reverse strategy, planned prolonged trigger exposure, might be more effective [6]. Analogously to fear-avoidance models, where short exposure to anxiety-provoking stimuli lead to increased anxiety responses, short exposure to headache triggers caused by their avoidance might result in a subsequent sensitization or reduced tolerance for the triggers [7]. In this way, recurrent headaches, migraine and other chronic pain conditions might develop and might be maintained in susceptible individuals. On the other side, prolonged exposure to triggers would lead to an increased tolerance through the process of adaptation and might reduce pain. Indeed, prolonged exposure to noise [8, 9], visual stimuli [10, 11] and stress [12] resulted in decreased nociceptive responses. Martin et al. suggested the behavioral management concept ‘Learning to Cope with Triggers’ (LCT) as preventive strategy [6, 13–15]. Here, triggers that were potentially harmful, e.g., hunger, dehydration or lack of sleep, should be avoided, but for other triggers, such as flicker or noise, prolonged exposure was used. Trigger exposures were accompanied by several methods of cognitive-behavioral therapy (education, management of dysfunctional thoughts, relapse prevention strategies) and relaxation training. When compared to an untreated waiting list, patients with recurrent headaches receiving LCT showed reduced headache and medication consumption, whereas for trigger avoidance combined with cognitive-behavioral therapy no effect was found [13, 15].

Besides the practice of coping skills, neurophysiological desensitization or habituation to the trigger is discussed as mechanism of effect of LCT. In migraine, a deficient interictal habituation has been reported for visual, somatosensory, and auditory evoked potentials (see [16] for review). Hereby, the initial response to a sensory stimulus seems normal or slightly lower, followed by an abnormal amplitude increase. This pattern does not correspond to response sensitization or to dishabituation but is characterized as lack of habituation. The same holds true for noxious stimuli, as demonstrated by contact-heat evoked potentials, laser-evoked potentials and the nociceptive blink reflex [16]. Attempts to normalize deficient habituation, potentially by planned exposure to migraine triggers, might thus be promising treatment strategy.

Following the controversy if triggers should be avoided or not, we compare two opposing treatment strategies for photophobia in migraine, light exposure and light deprivation. To this end, we investigated daily reports of ictal and interictal photophobia, presence and severity of headache and measured visual habituation during a flickering light task using functional magnetic resonance imaging (fMRI) in migraine patients and healthy controls. At baseline, we expected a lack of habituation in the primary visual cortex in the patients compared to the controls that is normalized after the light exposure intervention. In addition, we hypothesized that photophobia and headache symptoms are reduced after light exposure while no difference was expected for light deprivation. Further, we expected a correlation between visual habituation and clinical symptoms.

## Methods

### Participants

After screening 147 potential study participants, 10 patients with migraine (9 female, age = 28.70 ± 8.18 years) and 11 healthy controls (9 female, age = 23.73 ± 2.24 years) were included in this study. Inclusion criteria for the patients comprised (1) migraine without aura according to the criteria of the International Classification of Headache Disorders (ICHD-3 beta), 1–4 days with migraine per month in the preceding three months, a score between 2 and 6 on a numeric rating scale for the intensity of interictal photophobia (range from 0 to 10 (= max. intensity)) and (4) a score > 4 for ictal photophobia. Controls should have no personal or family history of migraine and a score < 2 for photophobia. To be included, participants had to be between 18 and 45 years of age and should not present current or previous circadian rhythm disorders, major depression, anxiety disorders or medication overuse. Besides migraine in the patient group, participants should not suffer from any other recurrent headache apart from infrequent tension-type headache. Migraine patients and healthy controls were recruited by notices on public billboards and newspaper articles. None of the participants received migraine prophylaxis treatment. From the 126 potential participants that were not eligible for the study, 36 had no interest after receiving detailed information, 26 were excluded due to psychiatric comorbidities, 25 were above 45 years of age, 10 patients scored below 2 in the photophobia score, 8 patients suffered from migraine with aura, 5 controls had a positive family history of migraine, 4 controls scored above 2 in the photophobia score, and for the rest other reasons impeded study participation.

### Study design

To compare two opposite treatment strategies for photophobia in migraine, participants spent one hour on 7 consecutive days exposed to flashing light (Flash) or darkness (Dark) using a randomized crossover design with a wash-out period of 3 months. During the Flash intervention, participants were seated 120 cm in front of a white curtain that was illuminated by an LED light source (Dawe stroboscope type 1214B, 5 Hz, Grossegger & Drbal). During the Dark intervention, participants were seated in the same room used for light exposure, but in complete darkness. The sequence of the interventions was randomly allocated (patients: Flash first (*N* = 6), Dark first (*N* = 4); controls: Flash first (*N* = 6), Dark first (*N* = 5)). One day before the first intervention, as well as one day after completing the Flash and Dark interventions, fMRI scans using a flickering light task were administered. The participants were asked to keep a diary 7 days before the first intervention, during and 7 days after the Flash and Dark interventions. The diary included presence of headache and interictal photophobia (numeric rating scale from 0 (no photophobia) to 10 (most severe photophobia)). If headache was present that day, questions regarding headache severity (mild (1), moderate (2), severe (3)) and ictal photophobia (0 – 10) had to be completed as well. Interictal photophobia represented the main outcome of this investigation. Headache frequency and functional activation in the primary visual cortex induced by flickering light served as secondary outcome measures. Further outcomes comprised headache severity and ictal photophobia as reported in the diary. To investigate a potential influence of medication and menstrual cycle, the participants were asked to note drugs used as abortive treatment and menses occurrence in the diary as well.

### MR imaging

MR measurements were performed using a 3 T SIEMENS PRISMA MR with a 64-channel head coil and comprised a T1-weighted structural image using a MPRAGE sequence (TE/TR = 2.7/1800 ms, inversion time = 900 ms, flip angle = 9°, resolution 1 mm isotropic), 10 functional runs with a flickering light task, and functional resting state scans before and after the flickering light task (data not reported here). For functional images, a T_2_*-weighted gradient-echo-planar imaging (EPI) sequence was applied, with 38 slices aligned to AC-PC, covering the whole brain including cerebellum (TE/TR = 30/2500 ms, flip angle = 90°, in-plane acceleration = GRAPPA 2, field of view = 230 × 230 mm, voxel size = 1.8 × 1.8 × 3 mm, 25% gap). For the flickering light task, a block design with 7 alternating blocks (20 s each) of flashing light (8 Hz) and darkness was used (140 s, 56 volumes per run).

### MR data analysis

**Fig. 1:**
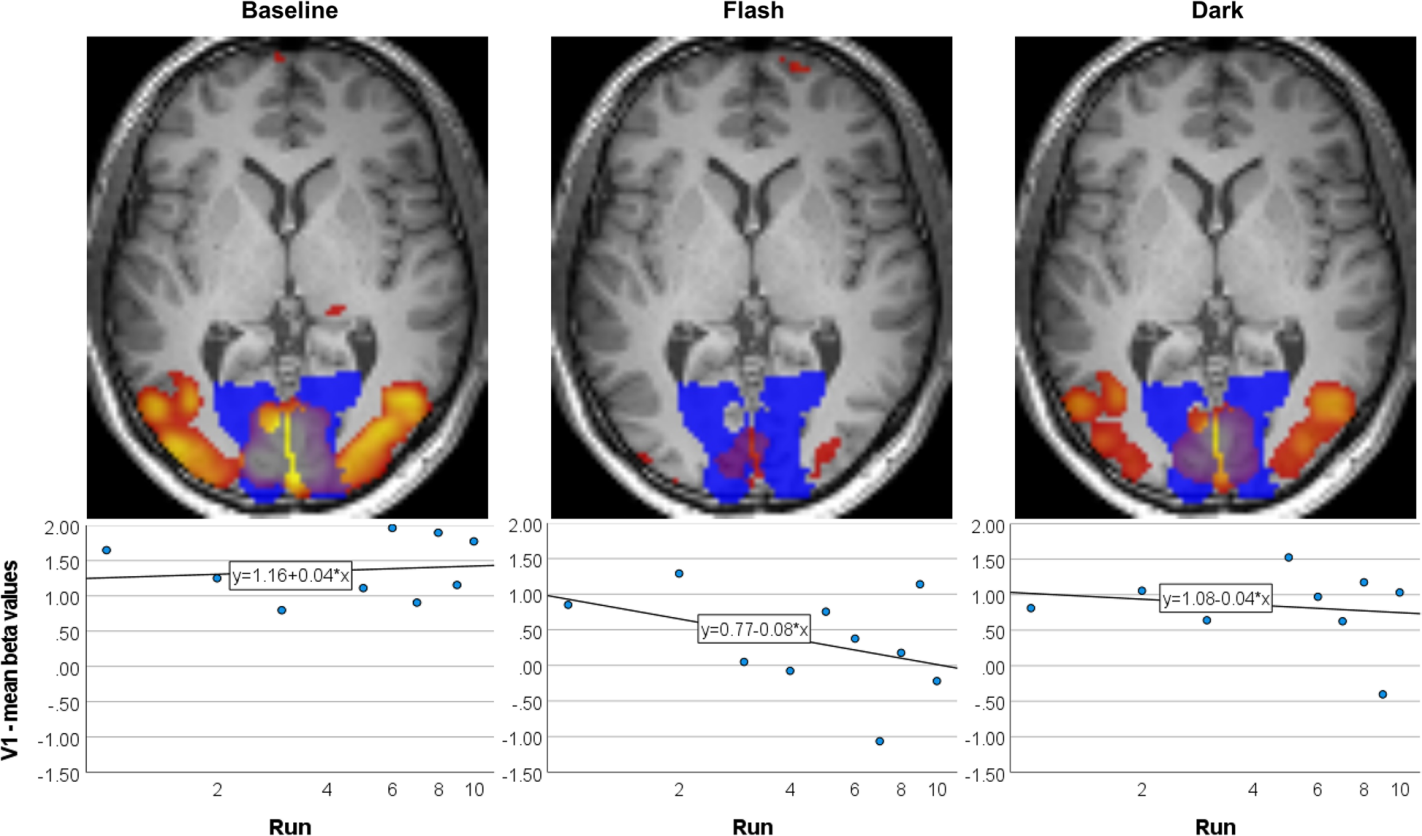
Individual habituation curves in the primary visual cortex (V1), illustrated for patient P07. Visual activation was induced by a flickering light task (10 runs, block design, FWE-corr. 0.05, activation displayed in red to yellow). Mean beta values within the V1 region of interest (blue) were extracted for each run and linearized using the natural log transform, resulting in uncorrected individual habituation curves per session

Data preprocessing, first and second level statistics were performed using SPM12 (https://www.fil.ion.ucl.ac.uk/spm/software/spm12). Data preprocessing comprised slice time correction, spatial realignment across all 3 fMRI sessions, coregistration of functional and structural images, structural segmentation, normalization to MNI (Montreal Neurological Institute) space and smoothing (6 mm FWHM Gaussian kernel). For first-level statistical analysis, a general linear model was used with the flashing light blocks convolved with the hemodynamic response function. A flexible factorial design with the between-subjects factor group (patients, controls), the within-subject factor condition (Baseline, Flash, Dark) was used on second level. To estimate within-subject effects, subject was included as factor in this model [17]. For analyzing differences relative to the baseline, the first-level contrasts Flash vs. Baseline and Dark vs. Baseline entered the flexible factorial second level model (subject and group as between-subjects factors, condition (Flash vs. Baseline, Dark vs. Baseline) as within-subject factor). For analyzing habituation within the primary visual cortex (V1, Brodmann’s area 17, calcarine cortex), mean beta values derived from the first-level analyses were extracted for the V1 region of interest (ROI) as defined by the cytoarchitectonic JuBrain Anatomy Toolbox [18] for each run of each session and subject (Fig. 1). The habituation slope for each session per subject was modeled using the regression: $Y=bX+a$. The mean ROI response (*Y*) is predicted by the natural log-transformed run number (*X*). The natural log transform linearizes the habituation curve, which is steepest during first runs, enabling linear regression analysis [19, 20]. As the habituation slope *b* is dependent on the initial amplitude *a* (the higher the initial amplitude, the higher the slope), the slopes were corrected according to Montagu (1963) [21]: b’ = b - c$\left(a-\overline{a}\right)$. Here, *b* is the individual regression slope, *a* the individual intercept (initial amplitude estimate), $\overline{a}$ represents the average group intercept and *c* the regression slope of b on a using all data. The individual corrected habituation slopes *b’* and initial amplitude estimates *a* entered repeated measurements ANOVAs with group as between-subject factor and time as within-subject factor using SPSS v26.

### Behavioral data analysis

Data analysis of the diary variables were performed using SPSS v26. One control had to be excluded due to missing data, resulting in a sample size of 10 for each group. Daily reports regarding presence and severity of headache, ictal and interical photophobia were averaged for the duration of each study phase (Baseline, Flash, Post Flash, Dark, Post Dark; 7 days each). As data was not normally distributed according to the Kolmogorov–Smirnov-Test, the non-parametric Friedman test for related samples was used for comparing the diary variables between the study phases. The effect of group was analyzed using the Quade nonparametric analysis of covariance with study phase as covariate and post-hoc Wilcoxon signed-rank tests (corrected for multiple comparisons using false discovery rate (FDR)). To test the relation between diary variables and V1 habituation estimates (*b’*, *a*), a non-parametric Spearman correlation analysis was applied for both groups separately and combined.

## Results

### Diary

**Fig. 2:**
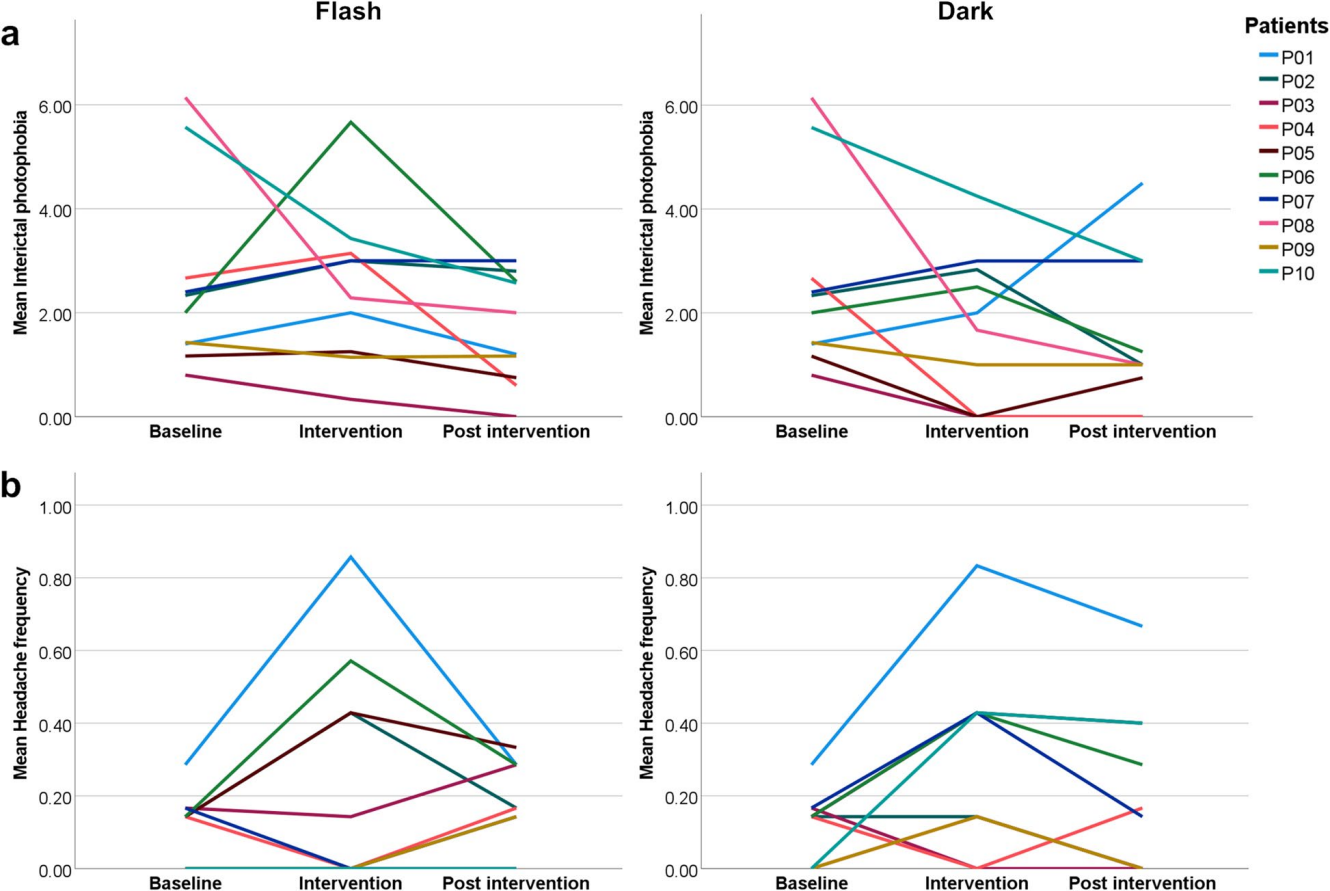
Mean interictal photophobia (**a**) and mean headache frequency (**b**) at baseline, during the intervention (Flash or Dark) and post intervention, plotted for all patients (P01-P10) individually. Interictal photophobia scores dropped after the Flash and after the Dark intervention in most of the patients. Regarding headache frequency, individual responses to the interventions were heterogenous but returned to baseline levels after the interventions

Group comparisons across all study phases were significant for headache frequency and severity, ictal and interictal photophobia (*p* < 0.001, Quade analysis of covariance), with significantly higher values for the patients compared to the controls (Table 1). Interictal photophobia was significantly higher in the patients compared to the controls in all study phases regardless of the intervention. During the Flash intervention and in the Post Dark period headache frequency and severity as well as ictal photophobia converge between groups, meaning that no significant effect of group was found here. In all other phases (Baseline, Dark, Post Flash) these variables were significantly higher in patients. Due to the significant group differences, the effect of study phase was analyzed for both groups separately, using non-parametric Friedman tests. At baseline, mean interictal photophobia was 2.59 (SD = 1.82) in the patients, while the controls scored close to 0 on average (mean = 0.06, SD = 0.18; Table 1). Interictal photophobia remained low in all study phases in the controls (< 0.02 on average) mirrored by a non-significant effect of study phase (*p* = 0.406). In the patients, interictal photophobia scores dropped during and after the Dark intervention, while the score remained high during the Flash intervention but dropped afterwards, which is also obvious in most patients individually (Fig. 2a). Accordingly, the Friedman test revealed a significant effect for study phase in the patients (*p* = 0.026) with a significant post-hoc comparison between Flash and Post Dark (*p* = 0.050 FDR-corr.). Further, we observed trends for lower values for the Post Dark vs. Baseline (*p* = 0.048 uncorr., *p* = 0.120 FDR-corr.), for Dark vs. Flash (*p* = 0.034 uncorr., *p* = 0.120 FDR-corr.) and for Post Flash vs. Flash (*p* = 0.048 uncorr., *p* = 0.120 FDR-corr.).

**Table 1:** Diary variables in the study phases for patients and controls

		**Interictal photophobia**		**Ictal photophobia**		**Headache frequency**		**Headache severity**	
		**Mean (SD)**	***P*-value (FDR-corr.)**^a^	**Mean (SD)**	***P*-value (FDR-corr.)**^a^	**Mean (SD)**	***P*-value (FDR-corr.)**^a^	**Mean (SD)**	***P*-value (FDR-corr.)**^a^
**Baseline**	**Patients (*N* = 10)**	2.59 (1.82)	0.002	2.85 (2.85)	0.012	0.12 (0.09)	0.025	1.15 (0.88)	0.023
	**Controls (*N* = 10)**	0.06 (0.18)		0.00 (0.00)		0.01 (0.05)		0.00 (0.00)	
	**Total (*N* = 20)**	1.32 (1.81)		1.43 (2.45)		0.07 (0.09)		0.58 (0.85)	
**Flash**	**Patients (*N* = 10)**	2.53^b^ (1.50)	0.002	2.02 (2.56)	0.079	0.24 (0.31)	0.190	0.71 (0.78)	0.247
	**Controls (*N* = 10)**	0.01 (0.05)		0.00 (0.00)		0.06 (0.14)		0.23 (0.50)	
	**Total (*N* = 20)**	1.27 (1.65)		1.01 (2.04)		0.15 (0.25)		0.47 (0.68)	
**Post Flash**	**Patients (*N* = 10)**	1.67 (1.06)	0.002	2.75 (2.42)	0.012	0.18 (0.12)	0.013	1.45 (0.96)	0.023
	**Controls (*N* = 10)**	0.00 (0.00)		0.00 (0.00)		0.02 (0.05)		0.20 (0.63)	
	**Total (*N* = 20)**	0.83 (1.12)		1.38 (2.18)		0.10 (0.12)		0.83 (1.02)	
**Dark**	**Patients (*N* = 10)**	1.73 (1.47)	0.007	3.11 (2.39)	0.012	0.30 (0.26)	0.013	1.21 (0.71)	0.025
	**Controls (N = 10)**	0.00 (0.00)		0.00 (0.00)		0.01 (0.05)		0.20 (0.63)	
	**Total (*N* = 20)**	0.86 (1.34)		1.56 (2.29)		0.16 (0.23)		0.70 (0.83)	
**Post Dark**	**Patients (*N* = 10)**	1.55^b^ (1.47)	0.089	2.48 (3.01)	0.003	0.21 (0.23)	0.111	1.10 (1.07)	0.247
	**Controls (*N* = 10)**	0.00 (0.00)		0.10 (0.32)		0.04 (0.07)		0.50 (0.85)	
	**Total (*N* = 20)**	0.78 (1.28)		1.29 (2.41)		0.12 (0.18)		0.80 (0.99)	

^a^Group comparisons using Quade analysis of covariance (adjusted for multiple comparisons using false discovery rate (FDR))

^b^Significant post-hoc comparisons between study phases (FDR-corr.)

The effect of study phase was not significant for headache frequency (patients: *p* = 0.261; controls: *p* = 0.722), headache severity (patients: *p* = 0.593; controls: *p* = 0.348) and for ictal photophobia (patients: *p* = 0.717; controls: *p* = 0.406). One reason for the insignificant results of these symptoms is probably the high variability in the individual responses to the interventions, as illustrated for headache frequency in Fig. 2b. Here, headache frequency increased in four patients, but dropped in three other patients during the Flash intervention compared to the baseline (three patients did not report headache at all). During the Dark intervention 7 patients reported increased headache compared to the baseline, while only two reported symptom decrease (one patient remained stable). After both interventions, headache frequency approximated baseline levels again.

Out of the 9 female patients, 7 noted a menses in the diary (one patient twice), on 23 days for all patients in total. As migraine tends to be more severe two days before and during menstruation [22]**,** headache occurrence was calculated for this time (39 days in total) and the remaining period (276 days). As headache was present on approximately 18% of the days before or during the menses and on approximately 20% of the remaining days, no significant modulation of headache frequency by the menstrual cycle was observed here. In total, patients reported headache on 76 days and used standard abortive medication (e.g., acetaminophen, triptans) in approximately half of the cases (on 37 days). The occurrence of headache varied considerably between the patients, for example, P01 reported headache on 22 days (9 with medication), P06 on 14 days (12 with medication), whereas P08 and P09 noted headache on two days only.

### Functional activation on group level

**Fig. 3:**
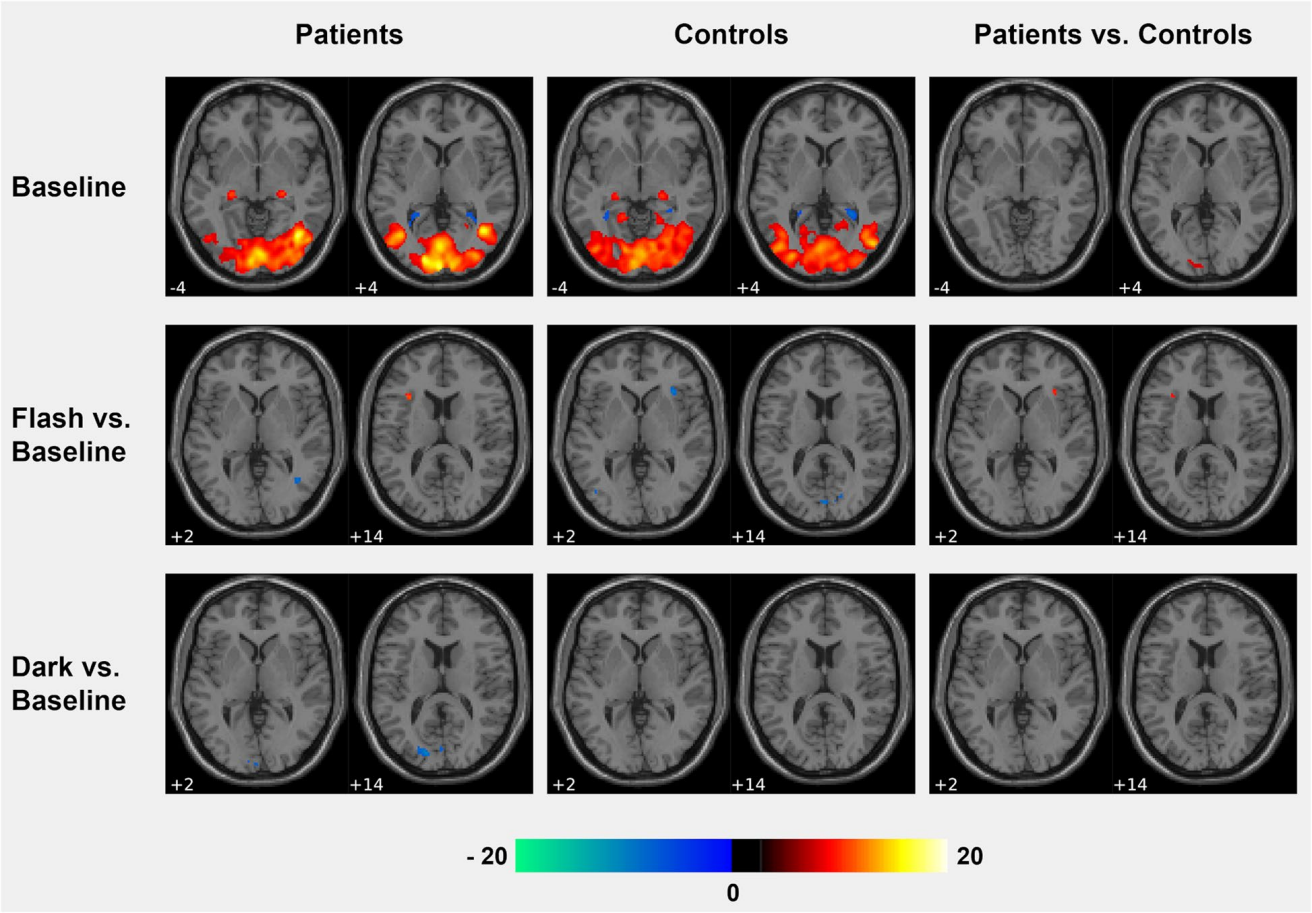
Functional activation induced by flickering light in patients and controls at baseline, and after Flash and Dark intervention, contrasted to the baseline (FWE 0.05 corr., cluster threshold = 10). At baseline, patients display higher activation in the primary visual cortex than controls. When contrasting Flash vs. baseline, patients show higher activation in the bilateral anterior insula compared to controls, while no difference was found for the contrast Dark vs. baseline

At baseline, both groups display widespread activation in occipital areas encompassing posterior temporal regions and in the lateral geniculate nucleus of the thalamus bilaterally (Fig. 3). In the controls, but not in the patients, right-hemispheric dominant fronto-parietal activation was observed as well. A contrast between patients and controls revealed significantly higher activation in the cerebellum and in the calcerine cortex in the patients while the activation in the left angular gyrus was lower compared to the controls. For the Flash intervention, the only significant difference was found in the anterior insula, with significantly higher activation in the patients compared to the controls. No significant effect of group was found for the Dark intervention. In Table 2 the significant cluster location with cluster size, MNI coordinates and highest T values are listed for the sessions separately and for contrasts between them.

**Table 2:** Significant light-induced activation at baseline, after Flash and Dark (FWE 0.05, k = 10)

					**Cluster peak MNI coordinates**		
		**Area (AAL)**	**T**	**k**	**x**	**y**	**z**
**Patients**	**Baseline**	Temporal- occ. Lobe bilat	18.6	12,455	40	-66	-6
		Thalamus (LGN) R	9.14	51	26	-24	-4
		Thalamus (LGN) L	10.82	67	-24	-24	-6
	**Flash**	Occ. Lobe bilat	12.62	4335	-2	-96	4
		MTG L	10.04	153	-42	-66	6
		Sup. Occ. R	7.87	43	26	-70	32
		Thalamus (LGN) R	7.66	19	24	-24	-4
		Inf. Occ. L	7.43	27	-36	-74	-12
		Thalamus (LGN) L	6.96	16	-22	-26	-6
		Sup. Occ. L	6.38	10	-20	-84	28
	**Dark**	Occ. Lobe bilat	12.85	3631	-14	-90	6
		MTG L	12.12	308	-42	-64	8
		Thalamus (LGN) L	9.38	47	-24	-26	-6
		Lingual L	9.07	195	-26	-86	-26
		Thalamus (LGN) R	9	38	24	-26	-4
		Sup. Occ. R	8.26	38	26	-68	30
		Fusiform L	6.76	51	-42	-56	-16
**Controls**	**Baseline**	Temporal- occ. Lobe bilat	14.75	13,416	48	-74	2
		Thalamus (LGN) L	9.31	61	-22	-26	-6
		Thalamus (LGN) R	8.77	52	22	-26	-4
		IFG R	8.08	96	32	8	28
		Precentral R	6.6	11	48	0	32
		SPL R	9.29	205	24	-58	46
		SPL L	8.03	47	-20	-56	48
	**Flash**	Occ. Lobe bilat	9.58	3828	-32	-88	-16
		Mid. Occ. L	8.23	112	-42	-80	0
		Thalamus (LGN) L	8.03	44	-24	-26	-6
		Calcerine R	7.04	27	18	-56	10
	**Dark**	Temporal- occ. Lobe bilat	12.83	6485	48	-74	2
		Mid. Occ. L	9.69	258	-42	-74	8
		Thalamus (LGN) L	8.47	55	-22	-26	-6
		Thalamus (LGN) R	7.45	27	24	-26	-6
		Sup. Occ. L	7.03	19	-20	-92	20
		Cuneus R	6.77	82	16	-82	26
**Patients vs. Controls**	**Baseline**	Cerebellum R	7.33	13	30	-80	-18
		Calcerine L	6.24	22	-4	-94	6
		ANG L	-6.65	12	-50	-74	24
	**Flash**	Insula R	7.49	47	36	24	4

Compared to the baseline measurement, patients showed higher activation in the Flash condition in the left insula and in bilateral inferior parietal areas, while activation was lower in regions dedicated to visual perception (right superior occipital, right fusiform gyrus and in the right posterior middle temporal lobe (visual area MT, Table 3). In the Dark condition, patients displayed higher activation in the right middle occipital gyrus, in the left supramarginal gyrus and in the right superior frontal gyrus and showed lower activation in several occipital regions and in the cerebellum compared to the baseline. In the controls, no area was found to be significantly higher in the Flash condition compared to the baseline. Lower activation was observed in several occipital regions, in the right anterior insula and in the right inferior frontal gyrus. In the Dark condition, controls displayed lower activation in the right inferior frontal gyrus compared to the baseline while the right superior frontal gyrus was significantly higher activated compared to the baseline. A direct comparison between patients and controls revealed significantly higher activation in the bilateral anterior insula in the patients for the Flash condition, while no significant difference between the groups was found for the Dark condition (Table 3).

**Table 3:** Significant light-induced activation relative to the baseline (FWE 0.05, k = 10)

					**Cluster peak MNI coordinates**		
		**Area (AAL)**	**T**	**k**	**x**	**y**	**z**
**Patients**	**Flash > Baseline**	SMG L	11.94	19	-44	-38	34
		Precentral R	10.35	12	48	-10	24
		SMG R	9.74	24	52	-28	24
		Insula L	9.68	20	-30	20	14
		IPL L	9.64	43	-32	-70	48
		ANG R	8.4	12	40	-62	50
	**Baseline > Flash**	Sup. Occ. R	12.36	64	28	-88	24
		MTG R	10.08	31	40	-62	4
		Fusiform R	9.17	38	26	-74	-4
	**Dark > Baseline**	SMG L	12.67	27	-44	-38	34
		Mid. Occ. R	9.05	24	44	-74	30
		SFG R	8.66	15	22	32	50
	**Baseline > Dark**	Sup. Occ. R	10.87	53	28	-88	26
		Cuneus L	10.4	67	-14	-86	14
		Sup. Occ. L	9.18	28	-4	-84	42
		Cerebellum R	9.12	12	26	-80	-20
		Cerebellum L	8.61	12	-18	-82	-22
		Calcerine L	8.42	32	-4	-96	-2
		Fusiform R	8.13	16	30	-78	-6
**Controls**	**Baseline > Flash**	IFG R	10.83	55	34	6	28
		Cuneus R	10.54	41	12	-82	18
		Insula R	9.65	41	34	24	2
		Lingual L	8.95	23	-16	-48	-4
		Calcerine L	8.89	18	2	-84	14
		Mid. Occ. L	8.79	19	-42	72	6
	**Dark > Baseline**	SFG R	8.64	11	22	34	50
	**Baseline > Dark**	IFG R	9.79	42	38	6	24
**Patients vs. Controls**	**Baseline > Flash**	Sup. Occ. R	12.36	64	28	-88	24
	**Flash > Baseline**	Insula R	8.63	15	34	24	2
		Insula L	8.69	17	-32	20	10

### Habituation in the primary visual cortex

**Fig. 4:**
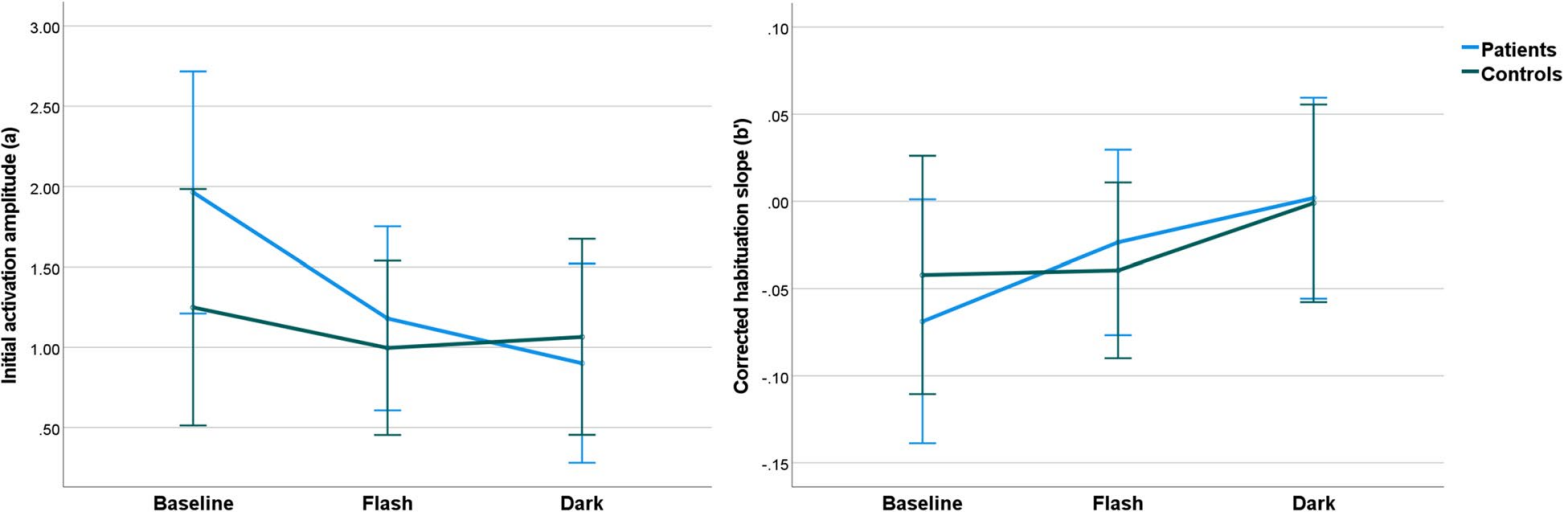
Initial activation amplitude (*a*) and corrected habituation slope (*b’*) in the patients and controls for the baseline, Flash and Dark intervention. Patients exhibited a significantly higher initial activation amplitude at baseline compared to Flash and Dark

Over all participants and sessions, the habituation slope of the beta values within the V1 ROI, as an estimate of primary visual cortex activation, is characterized by the regression:

$$Y= -0.02X+1.2Y$$

with the initial activation amplitude of *a* = 1.2 (= average group intercept) and the uncorrected regression slope *b* = -0.02. The regression analysis of all data with *b* as dependent variable and *a* as predictor resulted in the regression slope *c* = -0.044 that was used to calculate the corrected individual regression slopes *b’*. The average *a* and *b’* for both groups are plotted in Fig. 4. Repeated measurements ANOVAs with group as between-subject factor and time as within-subject factor for the corrected habituation slopes (*b’*) and the initial amplitude estimate (*a*) as dependent variables revealed no significant effects for *b’*, but for the initial amplitude a significant effect of condition (*p* = 0.002) and a significant interaction between condition and group (*p* = 0.042) was found. For both groups together, post-hoc comparison showed a significantly higher initial amplitude for the baseline compared to the Flash condition (*p* = 0.018, FDR-corr.) and compared to Dark (*p* = 0.027, FDR-corr.). For both groups separately, the comparisons showed significantly higher initial amplitude at baseline compared to Flash (*p* = 0.045, FDR-corr.) and compared to Dark (*p* = 0.045, FDR-corr.) in the patients. In the controls, only the comparison between baseline and Flash showed a trend for a lower initial amplitude (*p* = 0.081, FDR-corr.).

### Relation between diary reports and V1 habituation

**Fig. 5:**
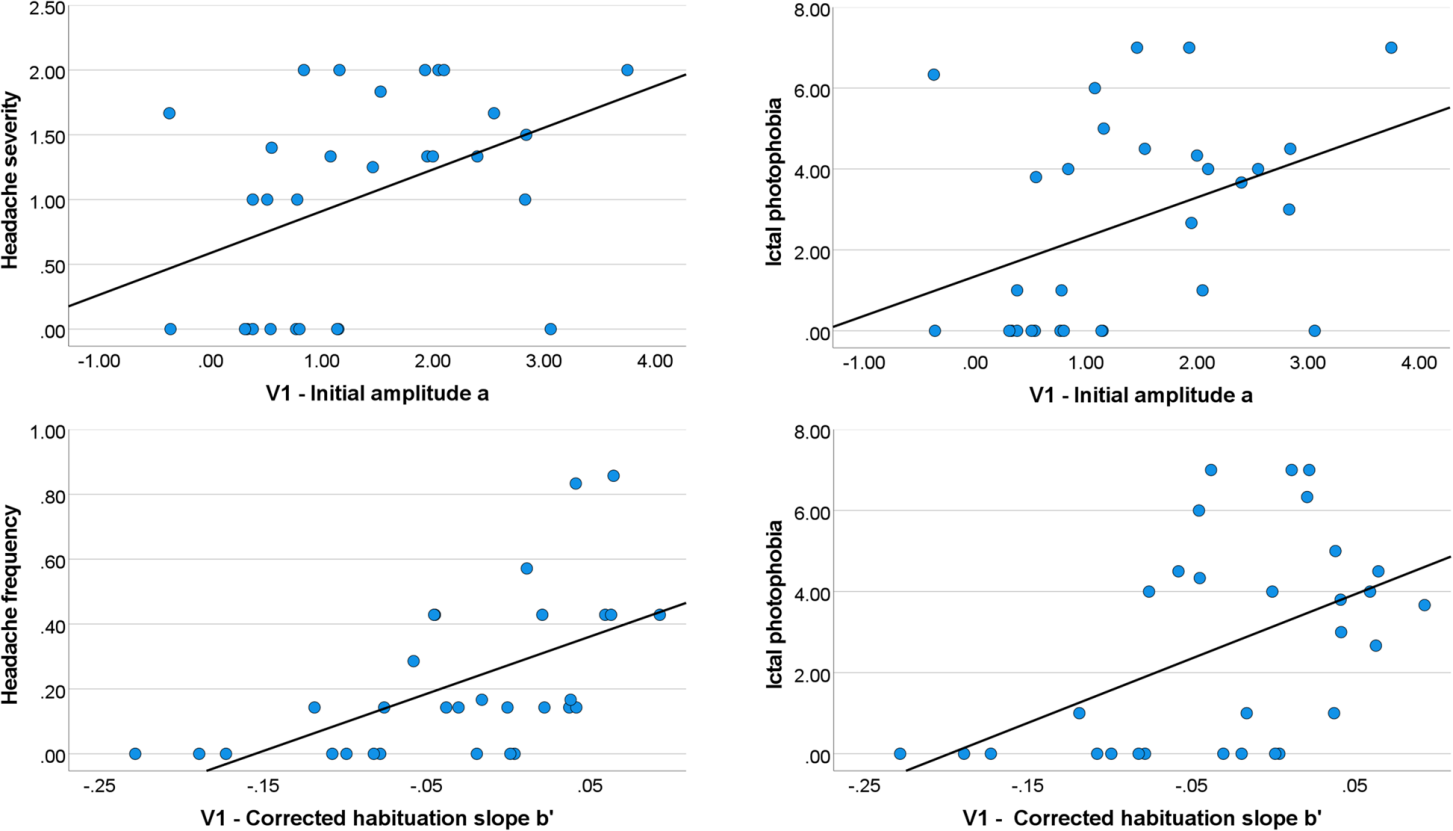
Correlations between primary visual cortex (V1) activation habituation estimates and diary variables in the patients. The initial amplitude was significantly correlated with headache severity and ictal photophobia, while the corrected habituation slope was significantly correlated with headache frequency and severity, and with ictal photophobia

Over all sessions and groups (*N* = 60), correlation analyses between diary variables and habituation slopes in the primary visual cortex revealed a significant correlation between the initial amplitude estimate *a* and headache severity (rho = 0.308, *p* = 0.034, FDR-corr.) and ictal photophobia (rho = 0.348, p = 0.024, FDR-corr.). In addition, *b’* was significantly correlated with headache frequency (rho = 0.378, *p* = 0.008, FDR-corr.) and severity (rho = 0.371, *p* = 0.008, FDR-corr.) as well as with ictal photophobia (rho = 0.289, *p* = 0.033, FDR-corr.). Analyzing patient data only (*N* = 30), resulted in even higher correlations (Fig. 5). The initial amplitude *a* significantly correlated with headache severity (rho = 0.469, *p* = 0.034, FDR-corr.) and ictal photophobia (rho = 0.432, *p* = 0.034, FDR-corr.). The corrected habituation slope b’ correlated significantly with headache frequency (rho = 0.651, *p* = 0.000, FDR-corr.), headache severity (rho = 0.538, *p* = 0.004, FDR-corr.) and with ictal photophobia (rho = 0.468, *p* = 0.012, FDR-corr.). For the controls (*N* = 30) no correlation turned out to be significant.

### Individual responses

**Fig. 6:**
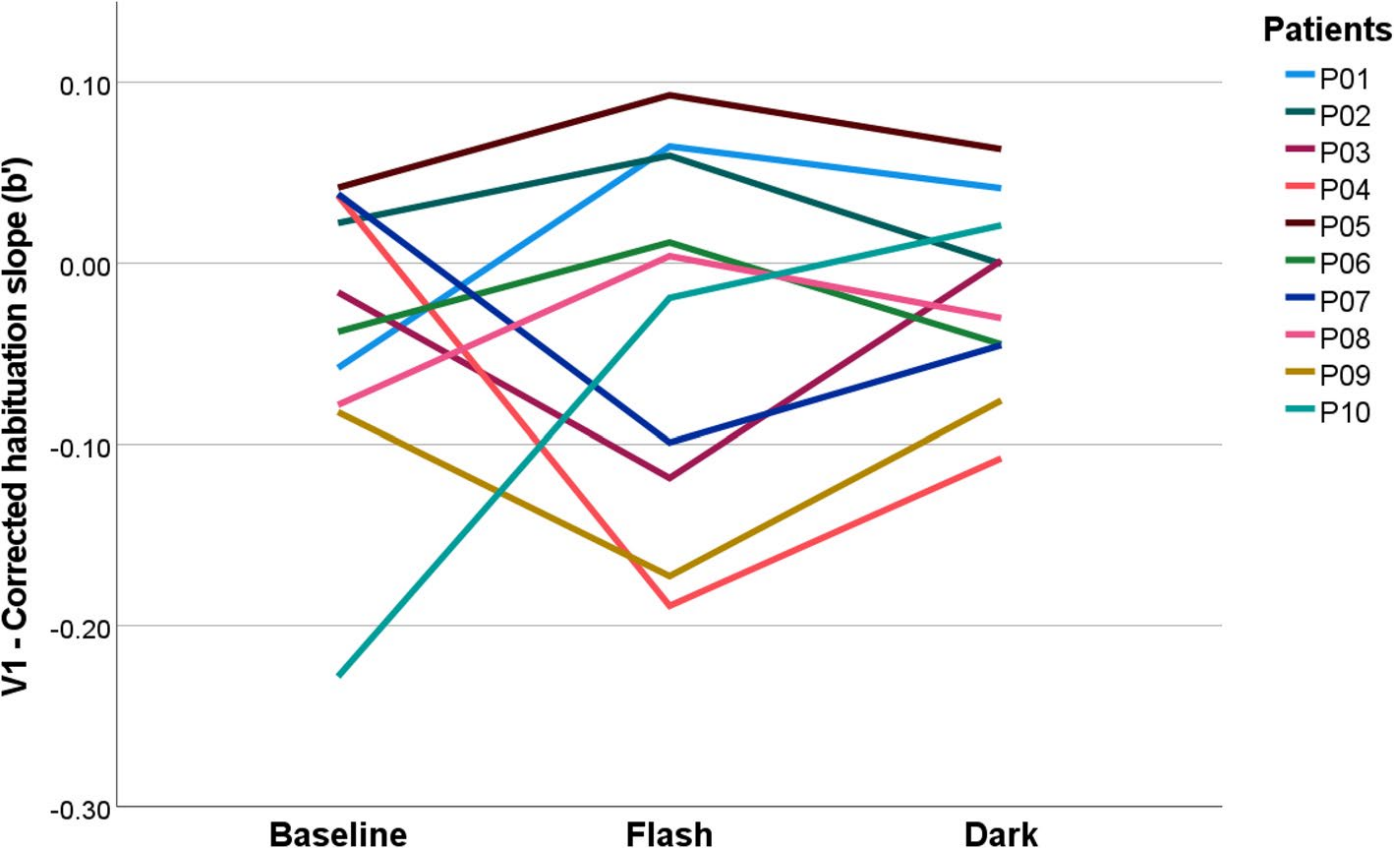
Corrected habituation slope (*b’*) for the primary visual cortex (V1) in all migraine patients individually. In the Flash condition, an increased habituation slope was found in 6 patients (P01, P02, P05, P06, P08, P10) and a decrease in 4 patients (P03, P04, P07, P09) compared to the baseline. In the Dark condition, 6 patients showed an increased *b’* (P01, P03, P05, P08, P09, P10) and 4 a declined *b’* (P02, P04, P06, P07) relative to the baseline

We found high variability in the individual course of the corrected habituation slope *b’*, particularly in the patients (Fig. 6). In relation to the baseline, 6 patients showed an increased *b’* (P01, P03, P05, P08, P09, P10) and 4 a declined *b’* (P02, P04, P06, P07) in the Dark condition. When comparing Flash to the baseline, an increased habituation slope was found in 6 patients (P01, P02, P05, P06, P08, P10) and a decrease in 4 patients (P03, P04, P07, P09). Interestingly, all patients reporting headache more frequently during the Flash intervention compared to the baseline (P01, P02, P05, P06, compare Fig. 2b) also revealed an increased habituation slope in the Flash compared to the baseline. Two further patients (P08, P10) showed an increased habituation slope, but had no difference in the headache frequency (no headache reported in the baseline and in the Flash condition). In line with these observations, all patients with a decreased headache frequency in the Flash intervention (P03, P04, P07), revealed a decreased habituation slope in the Flash compared to the baseline (as well as P09 who did not report headache at baseline or during the Flash intervention).

## Discussion

### Clinical symptoms

Not surprisingly, all clinical symptoms addressed in the diary, headache frequency and severity, ictal and interictal photophobia, were significantly more pronounced in the migraine patients than in the controls. It is important to bear in mind, that these obvious differences between the groups were enhanced by the inclusion and exclusion criteria for this study. The main study outcome interictal photophobia declined in the patients after both treatments compared to the baseline, but comparisons did not reach significance after correcting for multiple comparisons. We found significantly lower interictal photophobia in the Post Dark phase compared to the Flash intervention indicating a benefit for the Dark intervention. However, average values for interictal photophobia in the Post Flash phase were comparable to the Post Dark phase, so the treatment effect of the Flash intervention might only emerge after completing the treatment. No effect of study phase was found for headache frequency and severity or ictal photophobia, probably due to the high variability in the patients’ treatment responses. In the LCT approach, the trigger exposure treatment was conducted for 8 weeks and completed with cognitive-behavioral and relaxation techniques [13, 15]. With only one week, the duration of the interventions was probably too short to produce significant clinical improvements in the current investigation. Further, accompanying cognitive-behavioral support might be crucially for the treatment success of exposure therapy. Another important difference to previous LCT studies is that the current investigation primarily aimed to reduce photophobia. Thus, all patients in the current study had a moderate level of photophobia, but light discomfort was not necessarily a trigger for migraine attacks. Finally, the Dark intervention (1 h light deprivation) can be regarded as proper control condition for the Flash intervention (1 h light exposure), but it is not equivalent to trigger avoidance, where susceptible patients reduce light exposure throughout the day. During light deprivation, the participants might have tried to see something in the dark room, potentially causing eyestrain which is regarded as a form of visual disturbance [23]. As one hour in a room with complete darkness might not be a pleasant experience, the same coping strategies as during the uncomfortable light exposure might have taken place here. This would explain the reduction of photophobia after the Dark intervention, which contrasts our initial hypothesis.

### Functional imaging

Concerning functional activation induced by flickering light, we expected a lack of habituation in V1 in the patients at baseline. Indeed, we found an increased activation in occipital areas in the patients compared to the controls at baseline, compatible with a generally increased sensitivity to light in the patients and findings of previous fMRI studies [24]. Although the V1 habituation slope did not yield significant results, the initial activation amplitude was significantly higher at baseline compared to the measurements after both interventions in the patients. After the Flash and after the Dark intervention, initial V1 activation was at about the same level as in the controls, indicating a normalization of visual cortex excitability. This is also mirrored by lower activation in the bilateral temporo-occipital areas when directly contrasting the post interventions measurements with the baseline in the patients. In the controls, this pattern of reduced visual activation after the interventions was not found. Instead, we observed predominantly right-hemispheric fronto-parietal activation at baseline and increased activation in the inferior frontal gyrus (IFG) when comparing the post intervention sessions with the baseline. Interestingly, these regions are involved in cognitive reappraisal [25], anticipation and cognitive modulation of pain [26], indicating that controls might have used intrinsic cognitive control strategies to cope with the rather unpleasant flickering light, already during the baseline measurement. Another intriguing difference between groups was found for the anterior insula, a key region in the processing of emotionally salient stimuli [27], including pain [28]. Here, the only difference between groups emerged for baseline-corrected comparisons, with significantly higher activation in the bilateral anterior insula in the patients in the baseline-corrected Flash measurement. Correspondingly, a significantly higher activation was found in the anterior insula in the Flash condition relative to the baseline in the patients, while the opposite pattern, higher activation at baseline compared to Flash, was found in the controls. These findings indicate that patients perceived the flickering light after the Flash intervention as more salient, eventually even more painful, while for the controls the emotional relevance of this stimulus alleviated after the Flash intervention compared to the baseline.

### V1 habituation and headache symptoms

In accordance with our initial hypothesis, we found significant correlations between the habituation of V1 activation induced by flickering light and the clinical symptoms in the patients. The initial amplitude *a* significantly correlated with headache severity and ictal photophobia suggesting that more severe symptoms during the attacks were related to higher visual cortex excitability. The corrected habituation slope *b’* correlated significantly with headache frequency, headache severity and ictal photophobia indicating that better adaption to repeated flickering light stimulation is associated with fewer and milder headaches. Intriguingly, all patients reporting less headache in the week during the Flash intervention showed an increased habituation (steeper negative habituation slope *b’*) after the Flash intervention, compared to the baseline. The opposite was true as well, all patients suffering from more migraine attacks during the Flash intervention displayed impaired V1 habituation compared to the baseline. One might speculate that headaches generally lead to a decreased ability of the visual system to habituate, but if true, this should also account for the Dark intervention. Here, the correspondence between headache frequency and habituation slope in relation to the baseline was not as convincing (seven patients reported more headache, among them five patients with increased habituation slope, two patients reported less headache, one of them with decreased habituation slope). Thus, the Flash intervention might have led to better tolerance of light-induced discomfort in some patients that might have reduced their headaches as well. However, in the week after the Flash intervention, headache frequency returned to baseline levels, indicating that potential treatment effects could not be maintained. In other patients, light exposure therapy in this form was not adequate to increase habituation to light and to reduce headaches. We did not find characteristics (age, medication use, menstrual cycle) that could explain why certain individuals responded to the interventions and others did not. One reason might be that not all symptoms of photophobia have the same underlying cause, mirrored by the high variability in the structural and functional organization of visual pathways in people affected by migraine [29, 30]. In this study, we investigated individuals with episodic migraine, ranging from 2 to 22 days with migraine attacks during the observation period of 5 weeks. Potentially, the interventions are more beneficial for individuals with chronic migraine, as headaches but also photophobia are more pronounced here [31].

### Limitations

Besides the high variability in the treatment responses, the lack of significant change in clinical symptoms might also be due the small sample size. After screening 147 participants, only 21 (10 patients) were eligible and willing to participate in this study. Particularly the high rate of comorbid psychiatric conditions [4] resulted in the exclusion of several potential participants in the patient group. Several patients and controls did not meet the criteria regarding age, clinical symptoms or photophobia and a high number of potential participants regarded study participation as too effortful. Nevertheless, the within-subject design with its benefits regarding statistical power, can potentially compensate small sample size. In addition, strict in- and exclusion criteria were used to promote sample homogeneity improving statistical power as well. However, the exclusion criteria regarding psychiatric comorbidities and photophobia might have led to a selection bias, potentially limiting generalizability of the results. Due to the nature of the interventions, no blinding was possible, but both Flash and Dark interventions were presented as equivalently treatment options.

## Conclusions

On average, light exposure did not lead to symptom relief, potentially due to the short duration of the intervention and the high variability of the patients’ responses to the interventions. However, the strong relationship between visual cortex habituation and headache symptoms and its modulation by light exposure therapy might shed light on the neurophysiological basis of exposure treatment effects. The results encourage follow-up studies on behavioral treatments in migraine and the use of fMRI measuring individual neuronal habituation to repeated stimuli to monitor treatment efficacy of exposure therapy in chronic pain or anxiety disorders. 

## Data Availability

The datasets used and/or analyzed during the current study are available from the corresponding author on reasonable request.

## Abbreviations

ANG: Angular gyrus
Bilat: Bilateral
FDR: False discovery rate
fMRI: Functional magnetic resonance imaging
FWE: Familywise error rate
IFG: Inferior frontal gyrus
Inf.: Inferior
IPL: Inferior parietal lobe
L: Left
LCT: Learning to cope with triggers
LGN: Lateral geniculate nucleus
Mid.: Middle
MTG: Middle temporal gyrus
Occ.: Occipital
R: Right
ROI: Region of interest
SFG: Superior frontal gyrus
SMG: Supramarginal gyrus
Sup.: Superior
V1: Primary visual cortex
